# Real-world impact of COVID-19 vaccination, household exposure, and circulating SARS-CoV-2 variants on infection risk and symptom presentation in a U.S./Mexico border community

**DOI:** 10.3389/fpubh.2025.1497390

**Published:** 2025-06-09

**Authors:** Ariel Cohen, Breanna Reyes, Maria Linda Burola, Angel Lomeli, Arleth A. Escoto, Linda Salgin, Borsika A. Rabin, Nicole A. Stadnick, Ilya Zaslavsky, Robert Tukey, Louise C. Laurent, Marva Seifert

**Affiliations:** ^1^University of California San Diego, La Jolla, CA, United States; ^2^Department of Obstetrics, Gynecology and Reproductive Sciences, University of California San Diego, La Jolla, CA, United States; ^3^San Ysidro Health, Precision Park Lane, San Diego, CA, United States; ^4^Herbert Wertheim School of Public Health and Human Longevity Science, University of California San Diego, La Jolla, CA, United States; ^5^Altman Clinical and Translational Research Institute Dissemination and Implementation Science Center, University of California San Diego, San Diego, CA, United States; ^6^Department of Psychiatry, University of California San Diego, La Jolla, CA, United States; ^7^Child and Adolescent Services Research Center, San Diego, CA, United States; ^8^San Diego Supercomputer Center, University of California San Diego, La Jolla, CA, United States; ^9^Department of Pharmacology, University of California San Diego, La Jolla, CA, United States; ^10^Division of Pulmonary, Critical Care, and Sleep Medicine, Department of Medicine, University of California San Diego, La Jolla, CA, United States

**Keywords:** household exposure, COVID-19 vaccination, COVID-19 positivity, underserved Latino community, SARS-CoV-2 Variants

## Abstract

**Background:**

San Ysidro, a densely populated primarily Latino community near the U.S./Mexico border, reported the highest rate of COVID-19 infection in San Diego County. In this increased infection risk environment, we explored the impact of COVID-19 vaccine status, household exposure, and primary circulating SARS-CoV-2 variant on the probability of infection and symptom presentation while controlling for temporal and sociodemographic factors.

**Methods:**

Data were collected as part of CO-CREATE (Community-Driven Optimization of COVID-19 Testing to Reach and Engage underserved Areas for Testing Equity), a collaborative implementation study between University of California San Diego, a local Federally Qualified Health Center, and the Global Action Research Center. Self-reported sociodemographic factors, household exposure, vaccine status, and symptoms were extracted from a cross-sectional questionnaire completed by participants; PCR test results were used for analysis. Multi-level logistic regression, to account for repeat testing, was used to estimate the impact of self-reported vaccination status on COVID-19 household transmission. Logistic regression was used to characterize symptoms associated with predominate circulating SARS-CoV-2 variants.

**Results:**

Between May 2021 and March 2023, 11,412 PCR test results from 6,032 participants were analyzed. Individuals who were vaccinated and had a household exposure were 3.5 times [aOR 3.5 (95% CI 2.7, 4.6)] more likely to be PCR positive compared to individuals who were vaccinated and reported no household exposure; and individuals who were unvaccinated and reported a household exposure were 9.1 times [aOR 9.1 (95% CI 5.3, 15.5)] more likely to be PCR positive compared to individuals who were vaccinated and reported no household exposure. These results were obtained after adjusting for variant wave, age, language spoken, previous infection status, symptom status, and employment. Symptoms varied by predominate circulating SARS-CoV-2 variant, highlighting the impact of variant on disease presentation and potential vaccine efficacy.

**Conclusion:**

COVID-19 vaccination was associated with a reduced risk of COVID-19 infection after household exposure, highlighting the importance of equitable access to COVID-19 vaccines, specifically in communities experiencing higher infection rates. Our findings also underscore the necessity for enhancing workplace safety protocols, addressing language-specific considerations, and being cognizant of differing symptom presentation by variant.

## Background

Latino communities have consistently been disproportionately impacted by the COVID-19 pandemic; individuals who identify as Latino(a) have been overrepresented in cumulative case rates and experienced worse outcomes compared to members of other racial/ethnic minority groups (1). Nationally, COVID-19 diagnosis rates were higher in predominately Latino counties compared to non-Latino counties (90.9 vs. 82.0 per 100,000) (2). And, in California, an analysis of COVID-19 test results found that Latinos(as) were 8.1 times more likely to reside in high-exposure-risk households compared to individuals who identified as non-Latino(a) (1).

Systematic reviews of clinical trials and observational studies, along with household transmission studies, have demonstrated that COVID-19 vaccination not only reduces disease severity in individuals who are vaccinated but also reduces an individual’s risk of becoming infected (3–6). In addition, real-world evidence has begun to emerge, demonstrating the efficacy of COVID-19 vaccines in preventing household transmission (7, 8). However, there is currently a notable gap in research regarding the real-world impact of COVID-19 vaccination among ethnically marginalized and medically underserved Latino communities where high rates of COVID-19 transmission and dense housing conditions have been identified. Given the known elevated risk of COVID-19 exposure and infection in disadvantaged communities, it is essential to evaluate real-world COVID-19 vaccination efficacy in reducing household transmission at the individual level (9).

In order to address this gap, we leveraged data from a community-driven COVID-19 testing program to estimate the impact of self-reported vaccination status on COVID-19 household transmission in San Ysidro, a predominately (93%) Latino U.S./Mexico border community (10). During the pandemic, San Ysidro experienced the highest COVID-19 case rates of any San Diego County zip code (11). We also included each predominately circulating variant in our analysis to ensure we adjusted for highly transmissible SARS-CoV-2 variants in our evaluation of vaccine effectiveness (12).

Data collected on self-reported household exposure, vaccination status, temporal and sociodemographic factors, and COVID-19 PCR test results were used to better understand the real-world effectiveness of COVID-19 vaccination on transmission and symptom presentation in this under-resourced Latino community, using three distinct analytical approaches. First, the association between individual COVID-19 infection and various participant characteristics and temporal factors were assessed. Next, we evaluated the impact of COVID-19 vaccination on the risk of COVID-19 infection after household exposure. And finally, we quantified the odds of reported symptoms by predominate circulating SARS-CoV-2 variant.

## Methods

The Community-driven Optimization of COVID-19 testing to Reach and Engage underserved Areas for Testing Equity (CO-CREATE) study was a two-year implementation project, funded by the National Institutes of Health (NIH) Rapid Acceleration of Diagnostics for Underserved Populations (RADx-UP) program. The CO-CREATE study was designed to provide culturally acceptable testing services in partnership with the community. The University of California San Diego (UCSD) along with the Global Action Research Center and a Federally Qualified Health Center (FQHC) worked together to co-create an accessible COVID-19 testing program in the community of San Ysidro, with an emphasis on ensuring access for children, pregnant women, and their families (13–15).

### Study design

The CO-CREATE study methods have been described previously (13), however in brief, community members and patients of the partnering FQHC were invited to test with CO-CREATE either as part of a no-cost testing program or as part of the companion research study. Individuals of all ages were invited to participate, and those who opted to participate in the research study provided written informed consent. Exclusion criteria included the inability to provide assent, consent, or lack of a legal guardian capable of providing consent on the participants’ behalf. Participants were instructed by study staff to self-swab or, if necessary, swab with the assistance of a parent/guardian. Nasal swabs were couriered to the UCSD EXCITE clinical laboratory for SARS-CoV-2 PCR testing (EUA202755 and EUA210524) (13). Test results were returned within 1–3 days via the participant’s preferred method of contact, email or text, and all positive COVID-19 PCR results were followed up by a phone call from a clinician. Results were returned to participants in their preferred language as identified at registration (English or Spanish) (13).

Research study participants were invited to complete a questionnaire regarding their experience, exposure, knowledge, attitudes, and behaviors surrounding COVID-19 testing and vaccination. Questionnaires were comprised of Common Data Elements (CDEs) required by the RADx-UP program and additional site-specific questions (see Supplement Table S1) (16). Study participants who returned for additional testing were asked to complete a return questionnaire, which consisted of an abbreviated set of questions. Participants were compensated $20 USD for the initial survey and $10 USD for every return questionnaire completed.

### Data management

The CO-CREATE study questionnaire data were stored in REDCap, a HIPPA-compliant data collection software, and later accessed using REDCap Python API for analysis and alignment with NIH CDE vocabularies (13, 17). Additional self-reported data on COVID-19 vaccine status was captured by study staff at the time of participant enrollment and stored in a separate file used to track study enrollment. Participants were classified as having been vaccinated if they reported having received at least one COVID-19 vaccine when completing the questionnaire or at enrollment when questioned by study staff. Vaccine validation was not performed. COVID-19 household exposure was defined as having a household member with a COVID-19 diagnosis during the two weeks prior to testing. Participants were categorized into four mutually exclusive vaccine and exposure categories, vaccinated with no known household exposure (reference category), unvaccinated with no known household exposure, vaccinated with reported household exposure, and unvaccinated with reported household exposure. Age was aggregated into five categories, 0–11 years (reference category), 12–19 years, 20–39 years, 40–59 years, and 60 years plus. Individuals were classified as being Spanish speakers if they indicated they spoke Spanish at home. Being classified as employed included individuals who reported currently working versus individuals who reported being unemployed, looking for work, being retired, indicating they were students or preferred not to answer. Self-reported health status was categorized as those who indicated they had a health condition (e.g., diabetes, hypertension, or chronic obstructive pulmonary disease) versus those who did not indicate they had the condition.

The UCSD EXCITE lab performed the SARS-CoV-2 viral genome sequencing on PCR-positive samples to identify variant strains on a subset of samples (13). Sequencing results were used to identify predominate circulating SARS-CoV-2 variants during specific time periods and were categorized into ‘waves’.

### Study population

**Figure 1:**
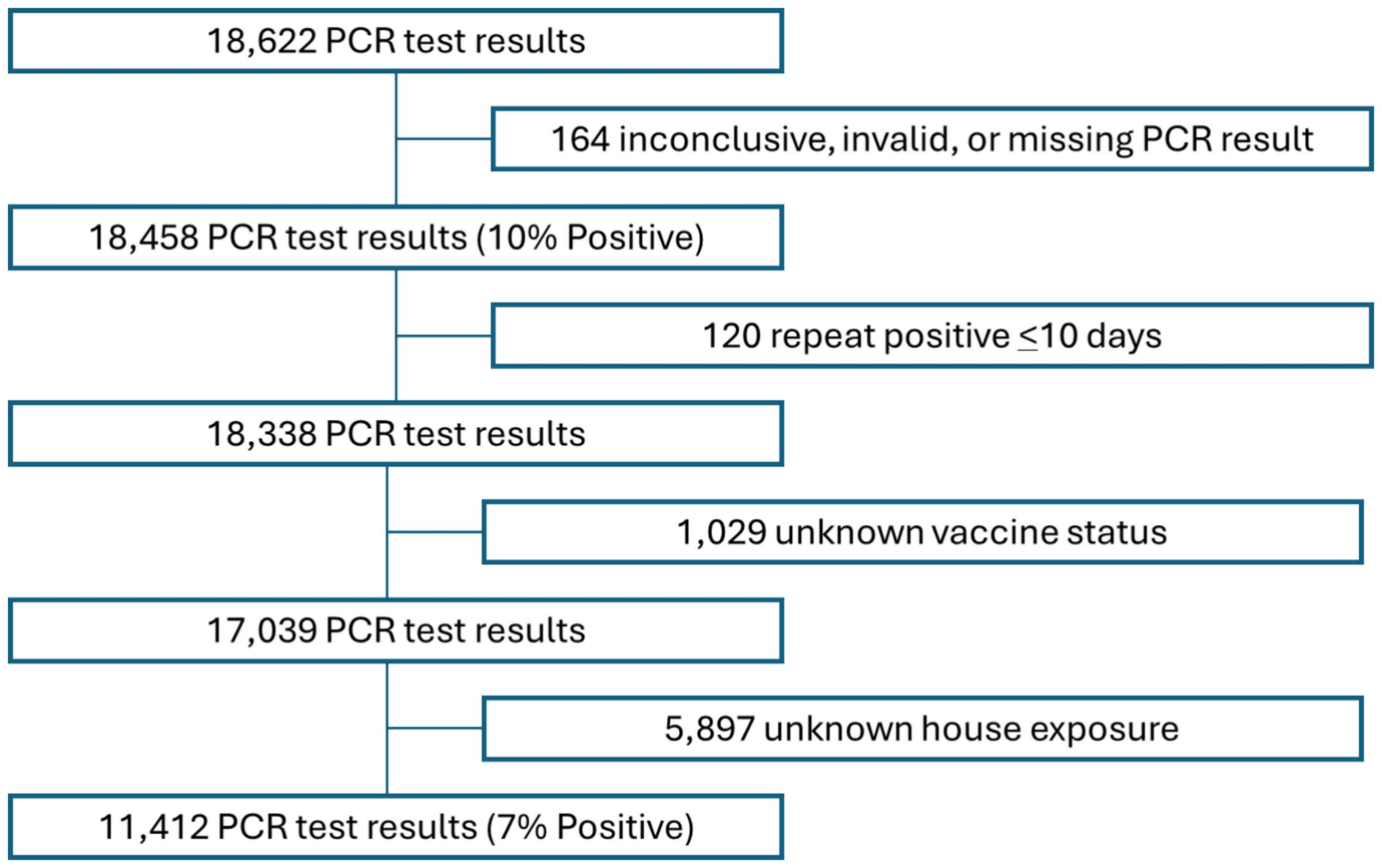
Schematic of COVID-19 results included in the analysis.

Between May 1, 2021, and March 31, 2023, 18,622 COVID-19 PCR tests were administered to study participants. Of all tests administered, 164 returned either inconclusive, invalid, or other results and were excluded from the analysis. In addition, 120 records with repeat positive test results, (i.e., tests that were administered less than 10 days after a previous positive test result), 1,029 records with missing vaccine status, and 5,897 records with unknown or missing history of COVID-19 household exposure data were excluded. The resulting 11,412 PCR test results records from 6,032 individuals (See Figure 1) were analyzed for this study.

### Statistical analysis

Three separate analyses were performed: (1) the association between individual COVID-19 infection and various participant characteristics and temporal factors was assessed, (2) the impact of COVID-19 vaccination on infection after household exposure was evaluated, and (3) the association of reported symptoms and COVID-19 infection was stratified by predominate circulating SARS-CoV-2 variant to characterize changes in disease presentation over time.

In detail, participant demographic and clinical characteristics were first evaluated by COVID-19 PCR result (SARS-CoV-2 detected versus not detected) at the individual test level and compared using Pearson’s X^2^. Second, a univariate analysis using a multi-level logistic regression to adjust for repeat testing was performed to identify participant-level factors associated with test positivity, individually and in a multivariable model. Next, Hosmer and Lemeshow’s approach to variable selection was used to construct a final multivariable multi-level logistic regression model with an interaction term (vaccination status times household exposure) to evaluate the association between vaccination status and household exposure with COVID-19 PCR test result. The adjusted odds ratios and 95% CIs were reported for all covariates. To evaluate symptom variation by predominate circulating SARS-CoV-2 variant at the time of swab collection, self-reported symptoms and COVID-19 PCR test result were assessed using logistic regression and stratified by specific time periods associated with dominant circulating SARS-CoV-2 variant. All statistical analyses were conducted using STATA17 (StataCorp, College Station).

## Results

### Factors associated with COVID-19 infection

Seven percent of all PCR tests evaluated returned a positive COVID-19 result. Eighty-two percent of included participants reported being vaccinated at the time of testing encounter and approximately 11% reported household exposure. Participant characteristics along with associated exposure, clinical data, and predominate circulating SARS-CoV-2 variant were compared to individual test result (see Table 1). At the individual test level, being exposed to a household member who had COVID-19, speaking Spanish, being employed, having had a previous COVID-19 infection, being older, reporting symptoms, and having a test date occur during specific circulating SARS-CoV-2 variants were factors significantly associated with a positive COVID-19 PCR result.

**Table 1:** Factors associated with positive PCR test results.

Characteristics, *n* (%)	Negative *n* = 10,614	Positive *n* = 798	Total *n* = 11,412	*p*-value
Vaccinated *	8715 (82.1)	642 (80.5)	9357 (82.0)	0.240
Known household contact exposure	1009 (9.5)	295 (37.0)	1,304 (11.4)	<0.001
Female sex	6,510 (61.3)	490 (61.4)	7000 (61.3)	0.969
Age				0.008
0–11	1166 (11.0)	90 (11.3)	1256 (11.0)	
12–19	891 (8.4)	90 (11.3)	981 (8.6)	
20–39	3223 (30.4)	248 (31.1)	3471 (30.4)	
40–59	3653 (34.4)	234 (29.3)	3887 (34.1)	
60+	1681 (15.8)	136 (17.0)	1817 (15.9)	
Spanish	9153 (86.2)	723 (90.6)	9876 (86.5)	<0.001
Employed	1729 (16.3)	272 (34.1)	2001 (17.5)	<0.001
Diabetes	1327 (12.5)	106 (13.3)	1433 (12.6)	0.521
Hypertension	1499 (14.1)	119 (14.9)	1618 (14.2)	0.538
Chronic obstructive pulmonary disease	209 (2.0)	10 (1.3)	219 (1.9)	0.155
Previously positive COVID	1364 (12.9)	282 (35.3)	1646 (14.4)	<0.001
Symptoms	4213 (39.7)	657 (82.3)	4870 (42.7)	<0.001
SARS-CoV-2 Variant ‘wave’				<0.001
5/1/21 to 12/21/21 (Gamma, P.1, Delta, B.1)	6421 (43.5)	169 (21.2)	4790 (42.0)	
12/22/21 to 4/4/22 (Omicron, BA.1)	1393 (12.1)	320 (40.1)	1713 (15.0)	
4/5/2022 to 7/6/22 (Omicron, BA.2)	1345 (12.7)	105 (13.2)	1450 (12.7)	
7/7/22 to 3/31/23 (BA.5, XBB)	3255 (30.7)	204 (25.6)	3459 (30.3)	

* Vaccinated includes those who self-reported at least one dose of a COVID-19 vaccine.

### Impact of vaccination on COVID-19 infection after household exposure

To assess the relationship between COVID-19 infection and self-reported household exposure in the context of vaccination status, we used multi-level logistic regression to adjust for multiple testing by individuals and evaluated each factor individually, then in a multivariable ‘full’ model, and finally in a ‘final’ model excluding variables that were not statistically significant in the full model (Table 2). In the final model, we found that being vaccinated and having a household exposure was significantly associated with a positive PCR result (aOR 3.51 [95% CI: 2.66, 4.64]) compared to individuals who were vaccinated and did not have a household exposure, after controlling for sex, age, language spoken, employment, previous infection, symptom presentation, and predominate SARS-CoV-2 variant at the time of testing (See Table 2). Among individuals who were not vaccinated and had a household exposure, the association with a COVID-19 PCR positive result was significantly higher [aOR 9.09 (95% CI: 5.34, 15.46)], suggesting that in the presence of household exposure, being vaccinated for COVID-19 reduced the likelihood of COVID-19 infection after controlling for language spoken, employment, age, having a previous infection with COVID-19, being symptomatic, and predominant circulating SARS-CoV-2 variant.

**Table 2:** Multi-level regression models evaluating the association between COVID-19 infection and household exposure accounting for vaccination status.

Characteristic	Univariate aOR (95% CI)	Full model aOR (95% CI)	Final model aOR (95% CI)
Exposure and vaccination			
Vaccinated with no household exposure (ref)			
Unvaccinated with no household exposure	0.75 (0.52, 1.05)	1.45 (1.03, 2.04)	1.48 (1.05, 2.08)
Vaccinated with household exposure	7.92 (5.80, 10.8)	3.51 (2.66, 4.64)	3.51 (2.66, 4.64)
Unvaccinated with household exposure	10.7 (6.24, 18.4)	9.13 (5.34, 15.05)	9.09 (5.34, 15.46)
Age category			
0–11 (ref)			
12–19	1.33 (1.09, 1.63)	1.54 (0.97, 2.45)	1.54 (0.97, 2.46)
20–39	2.29 (1.95, 2.68)	1.03 (0.68, 1.56)	0.99 (0.66, 1.50)
40–59	2.27 (1.94, 2.66)	1.10 (0.71, 1.69)	1.06 (0.69, 1.61)
60+	1.89 (1.59, 2.25)	1.73 (1.08, 2.79)	1.68 (1.06, 2.64)
Spanish speaker (vs other language speaker)	1.52 (1.35, 1.72)	1.48 (1.04, 2.10)	1.48 (1.03, 2.10)
Employed (vs not working, student, or unknown)	1.66 (1.49, 1.84)	1.96 (1.52, 2.52)	2.01 (1.56, 2.58)
Previous infection (vs no previous infection, missing)	4.01 (3.12, 5.17)	1.92 (1.51, 2.45)	1.92 (1.51, 2.44)
Symptoms (vs no symptoms)	12.1 (8.48, 17.2)	8.15 (6.12, 10.87)	8.10 (6.09, 10.78)
Predominant SARS-CoV-2 variant			
5/1/21 to 12/21/21 (Gamma, P.1 and Delta, B.1) (ref)			
12/22/21 to 4/4/22 (Omicron, BA.1)	25.1 (16.4, 38.3)	8.97 (6.41, 12.57)	9.01 (6.42, 12.64)
4/5/2022 to 7/6/22 (Omicron, BA.2)	7.25 (4.56, 11.5)	3.77 (2.21, 5.43)	3.77 (2.61, 5.43)
7/7/22 to 3/31/23 (BA.5 and XBB)	6.35 (4.19, 9.63)	3.44 (2.49, 4.69)	3.44 (2.51, 4.73)
Sex female (vs male, undefined, missing)	0.88 (0.80, 5.97)	1.18 (0.94, 1.48)	
Diabetes (vs no diagnosis, or missing)	1.59 (1.35, 1.87)	1.18 (0.83, 1.69)	
Chronic obstructive pulmonary disease (vs no diagnosis, or missing)	2.27 (1.51, 3.39)	0.55 (0.22, 1.38)	
Hypertension (vs no diagnosis, or missing)	1.58 (1.37, 1.84)	0.86 (0.61, 1.21)	

### Symptom presentation stratified by predominate circulating SARS-CoV-2 variant

Finally, we examined the association between self-reported symptoms and COVID-19 PCR test results by predominate circulating SARS-CoV-2 variant to assess changes in disease presentation by variant. We used the date of test collection as a proxy for SARS-CoV-2 variant based on previous sequence analysis indicating well-defined time periods for specific variant predominance (10). All positives reported between May 1, 2021, and December 21, 2021, were categorized as Gamma or Delta; positive results between December 22, 2021, and April 4, 2022, were categorized as Omicron; positive results between April 5, 2022, and July 6, 2022, were categorized as BA.2; and positive results between July 7, 2022, and March 31, 2023, were categorized as BA.4/5. Participants were excluded from this analysis if they did not indicate either the presence or absence of each reported symptom, resulting in 9,918 records for analysis.

**Figure 2:**
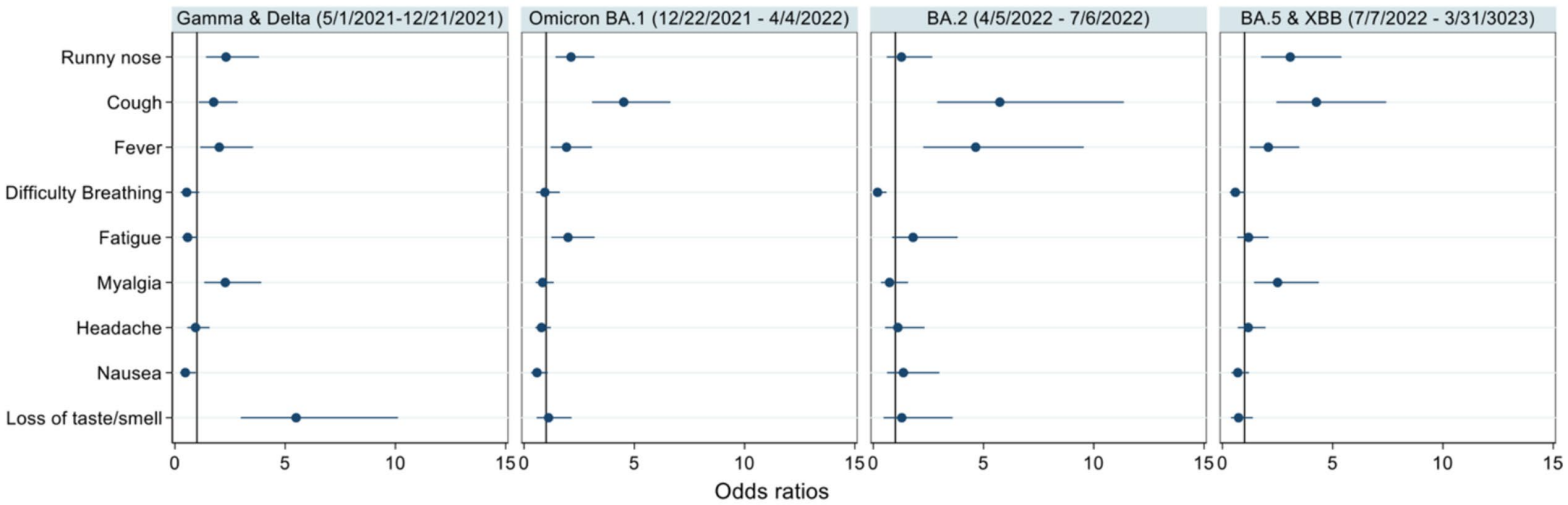
Odds ratios of self-reported symptoms among individuals who returned a positive COVID-19 PCR result. The odds of self-reporting each symptom among those who returned a positive COVID-19 result compared to those who did not return a positive result were stratified by date and associated variant wave. Point estimates are indicated by the dot and 95% confidence intervals are represented by the line.

During the Gamma and Delta waves, runny nose, cough, fever, myalgia, and loss of taste and smell were independently associated with positive COVID-19 PCR test results. During the Omicron BA.1 wave, runny nose, cough, fever, and fatigue were associated with positive COVID-19 PCR test results. During the Omicron BA.2 wave, cough and fever were associated with test positivity, and difficulty breathing appeared protective or inversely associated with positive COVID-19 PCR test results. Finally, during the Omicron BA 4/5 wave, runny nose, cough, fever, and myalgia were associated with test positivity (see Figure 2).

## Discussion

In this study, COVID-19 vaccination appeared to be protective and was associated with a reduced likelihood of positive COVID-19 PCR results after reported household exposure to COVID-19. This association persisted after accounting for various risk factors, including predominate variant type, age, primary language spoken, previous infection status, symptom presentation, and employment status. While previous studies have highlighted the effectiveness of COVID-19 vaccines in reducing household transmission (18–21), few studies have specifically examined the real-world impact of vaccination on COVID-19 infection after household exposure in Latino communities with previously identified risk factors including lack of health insurance, higher household density, and a greater proportion of monolingual Spanish-speaking or bilingual individuals (2).

Our analysis encompassed self-reported data collected during multiple SARS-CoV-2 variant waves and the association between vaccination and a reduced probability of returning a positive COVID-19 PCR result after household exposure remained consistent, even as the predominate circulating SARS-CoV-2 variants shifted. This finding is consistent with multiple studies, including a South Korean prospective cross-sectional study, which found the household secondary attack rates (SAR) for unvaccinated and vaccinated Omicron variant exposed contacts were 44 and 41% and, during the Delta wave, were 35 and 23% for unvaccinated and vaccinated exposed contacts (22). Similarly in the Netherlands, when the Delta variant was prevalent, full vaccination of the index case reduced transmission to unvaccinated and fully vaccinated household contacts by 63 and 40%; during the Alpha variant’s dominance, the SARs among household contacts were higher among unvaccinated compared to vaccinated contacts at 31 and 11%, respectively (23, 24). Our results indicate that COVID-19 vaccination lowers the risk of household transmission regardless of circulating SARS-CoV-2 variant.

While language itself is not biologically correlated with increased risk of COVID-19 infection, in our study, Spanish language preference is likely a proxy for other risk factors associated with COVID-19 positivity and has been identified in previous studies. In a Baltimore-based study assessing risk factors for COVID-19 infection in Latino communities, it was found that individuals who tested positive were more likely to report Spanish as their preferred language and have a larger household size compared to individuals who tested negative (25). Additionally, in California, it was found that limited English proficiency had the largest impact on the association between the proportion of Latino residents and COVID-19 cases (26). Furthermore, it was found that a higher risk of COVID-19 infection was associated with non-English language preference after adjusting for age, race/ethnicity, and social factors (27, 28). This association between low English proficiency/Spanish as a preferred language and increased COVID-19 infection risk is thought to be driven by factors such as difficulty finding COVID-19 testing sites, transportation issues, and language barriers were contributing factors (29). Our study’s findings along with the evidence of the association between language and COVID-19 infection underscores the crucial need for language-sensitive approaches to public health interventions and emphasizes the importance of ongoing vaccination campaigns, especially in underserved and vulnerable communities.

An additional strength of our study was the inclusion of employment status in our final model. Individuals employed, compared to those not employed, students, or those with missing employment status, exhibited higher odds of testing positive for COVID-19. This finding aligns with previous work which has determined that being employed along with employment type is associated with elevated COVID-19 infection rates (27, 30). Research suggests that greater work exposures likely contribute to a higher prevalence of COVID-19 infection among Latino(a) adults (31), emphasizing the critical need to equitably allocate Personal Protective Equipment and access to screening in Latino communities (30, 32).

While few studies have explored disease presentation by SARS-CoV-2 variant (33) it is understood that there are differences in clinical presentation by region, age, and co-morbidity (34). Our results indicate that as the predominate SARS-CoV-2 variant evolved, there were meaningful changes in the odds of symptom presentation among individuals infected with COVID-19. Our results demonstrated the marked decrease in loss of taste or smell as variants evolved, and in the increase in symptom presentation of cough, runny nose, fever, and myalgia. Our findings are consistent with previous work that demonstrated the decrease in loss of taste or smell and increase in cough during the Omicron variant dominance compared to Delta and Gamma variants (34–37).

## Strengths and limitations

The main study limitation was that vaccine status was self-reported. Proof of vaccination was not collected from study participants and information on the time interval between vaccination and testing was not calculated. However, previous studies have demonstrated that self-reported vaccine status correlates well with documented vaccine status for COVID-19 (38, 39). We acknowledge there was a potential for response bias, as participants may have felt obligated to claim vaccination when interacting with our health research team. However, by placing a high priority on anonymity, we maintain that this limitation did not significantly compromise the validity of our study and likely would have biased the results toward the null. Additional potential limitations included the lower participation rate of individuals who tested positive, potentially reducing power to evaluate symptoms associated with disease, and the reliance on self-reported demographic and clinical data. However, reliance on self-reported data and any resulting misclassifications would likely dilute the observed signal.

To ensure broad participation, we prioritized inclusive approaches to recruitment and study engagement. The primary testing site was co-located with a FQHC in San Ysidro, a predominantly Latino community, and we ensured accessibility by offering the questionnaire in English and Spanish (13). Our commitment to cultural competence included the hiring of bilingual staff which were members of the Latino community and success was demonstrated by the trust build within the community as evidenced by a high rate of repeat participants. Recognizing potential challenges for older participants with electronic questionnaires, we provided paper questionnaires and offered verbal questionnaires for individuals with visual impairments or low literacy (13). These concerted efforts not only highlight the strengths of our study but also played a pivotal role in maximizing participant engagement and questionnaire completion.

## Conclusion

While multiple factors were associated with increased risk of COVID-19 infection in our study population, COVID-19 vaccination appeared to reduce the risk of infection even after household exposure, across multiple SARS-CoV-2 variants. Further research is needed to elucidate specific transmission pathways and the most effective mitigating behaviors in vulnerable populations. Additionally, tailoring public health measures to the specific needs of communities is essential for reducing disproportional impacts of COVID-19 infection.

## Data Availability

The raw data supporting the conclusions of this article will be made available by the authors, without undue reservation.

## References

[1] Reitsma MB Claypool AL Vargo J Shete PB McCorvie R Wheeler WH. Racial/ethnic disparities in COVID-19 exposure risk, testing, and cases at the subcounty level in California. Health Aff. (2021) 40:870–8. doi: 10.1377/hlthaff.2021.00098 PMC8458028 33979192

[2] Rodriguez-Diaz CE Guilamo-Ramos V Mena L Hall E Honermann B Crowley JS. Risk for COVID-19 infection and death among Latinos in the United States: examining heterogeneity in transmission dynamics. Ann Epidemiol. (2020) 52:46–53.e2. doi: 10.1016/j.annepidem.2020.07.007 32711053 PMC7375962

[3] Pormohammad A Zarei M Ghorbani S Mohammadi M Razizadeh MH Turner DL. Efficacy and safety of COVID-19 vaccines: a systematic review and meta-analysis of randomized clinical trials. Vaccines. (2021) 9:467. doi: 10.3390/vaccines9050467 34066475 PMC8148145

[4] Graña C Ghosn L Evrenoglou T Jarde A Minozzi S Bergman H. Efficacy and safety of COVID-19 vaccines. Cochrane Database Syst Rev. (2022) 2023:3. doi: 10.1002/14651858.CD015477, PMID: 36473651 PMC9726273

[5] Deplanque D Launay O. Efficacy of COVID-19 vaccines: from clinical trials to real life. Therapies. (2021) 76:277–83. doi: 10.1016/j.therap.2021.05.004 PMC8114590 34049688

[6] Harder T Koch J Vygen-Bonnet S Külper-Schiek W Pilic A Reda S. Efficacy and effectiveness of COVID-19 vaccines against SARS-CoV-2 infection: interim results of a living systematic review, 1 January to 14 may 2021. Euro Surveill. (2021) 26:2100563. doi: 10.2807/1560-7917.ES.2021.26.28.2100563, PMID: 34269175 PMC8284046

[7] Shah Anoop SV Gribben C Bishop J Hanlon P Caldwell D Wood R. Effect of vaccination on transmission of SARS-CoV-2. N Engl J Med. (2021) 385:1718–20. doi: 10.1056/NEJMc2106757, PMID: 34496200 PMC8451182

[8] Maeda M Murata F Fukuda H. Effect of COVID-19 vaccination on household transmission of SARS-CoV-2 in the omicron era: the vaccine effectiveness, networking, and universal safety (VENUS) study. Int J Infect Dis. (2023) 134:200–6. doi: 10.1016/j.ijid.2023.06.017 37356650 PMC10289267

[9] Hu S Xiong C Zhao Y Yuan X Wang X. Vaccination, human mobility, and COVID-19 health outcomes: empirical comparison before and during the outbreak of SARS-Cov-2 B.1.1.529 (omicron) variant. Vaccine. (2023) 41:5097–112. doi: 10.1016/j.vaccine.2023.05.056 37270367 PMC10234469

[10] Reyes BJ Calvillo ST Escoto AA Lomeli A Burola ML Gay L. Community utilization of a co-created COVID-19 testing program in a US/Mexico border community. BMC Public Health. (2024) 24:3194. doi: 10.1186/s12889-024-20527-4 39558266 PMC11572090

[11] COVID-19 Cases by Geography of Residence. (2025). Available online at: https://www.sandiegocounty.gov/content/sdc/hhsa/programs/phs/community_epidemiology/dc/2019-nCoV/status/COVID19_Cases_by_Geography_of_Residence.html (Accessed March 13, 2025).

[12] Carabelli AM Peacock TP Thorne LG Harvey WT Hughes J de Silva TI. SARS-CoV-2 variant biology: immune escape, transmission and fitness. Nat Rev Microbiol. (2023) 21:162–77. doi: 10.1038/s41579-022-00841-7, PMID: 36653446 PMC9847462

[13] Lomeli A Escoto AA Reyes B Burola MLM Tinoco-Calvillo S Villegas I. Factors associated with COVID-19 vaccine uptake in a US/Mexico border community: demographics, previous influenza vaccination, and trusted sources of health information. Front Public Health. (2023) 11:11. doi: 10.3389/fpubh.2023.1163617 PMC10415906 37575117

[14] Salgin L Ayers LO Burola M-L Engler A-M Osuna A Gay L. Perceived COVID-19 risk and testing experiences in the san Ysidro U.S./Mexico border region. Transl Behav Med. (2023) 13:432–41. doi: 10.1093/tbm/ibac120 36999822 PMC10314726

[15] Stadnick NA Cain KL Oswald W Watson P Ibarra M Lagoc R. Co-creating a theory of change to advance COVID-19 testing and vaccine uptake in underserved communities. Health Serv Res. (2022) 57:149–57. doi: 10.1111/1475-6773.13910 35243622 PMC9108217

[16] Korzekwinski K. RADx-UP linkage datasets. RADx-UP. (2023) 76:277–83. Available online at: https://radx-up.org/resource-type/research-tools/radx-up-linkage-datasets/

[17] Harris PA Taylor R Thielke R Payne J Gonzalez N Conde JG. Research electronic data capture (REDCap)—a metadata-driven methodology and workflow process for providing translational research informatics support. J Biomed Inform. (2009) 42:377–81. doi: 10.1016/j.jbi.2008.08.010 18929686 PMC2700030

[18] Zheng C Shao W Chen X Zhang B Wang G Zhang W. Real-world effectiveness of COVID-19 vaccines: a literature review and meta-analysis. Int J Infect Dis. (2022) 114:252–60. doi: 10.1016/j.ijid.2021.11.009 34800687 PMC8595975

[19] Peters LL Raymer DS Pal JD Ambardekar AV. Association of COVID-19 vaccination with risk of COVID-19 infection, hospitalization, and death in heart transplant recipients. JAMA Cardiol. (2022) 7:651–4. doi: 10.1001/jamacardio.2022.0670 35475896 PMC9047723

[20] White Elizabeth M Yang X Blackman C Feifer Richard A Gravenstein S Mor V. Incident SARS-CoV-2 infection among mRNA-vaccinated and unvaccinated nursing home residents. N Engl J Med. (2021) 385:474–6. doi: 10.1056/NEJMc2104849 34010526 PMC8174028

[21] Marcelin JR Pettifor A Janes H Brown ER Kublin JG Stephenson KE. COVID-19 vaccines and SARS-CoV-2 transmission in the era of new variants: a review and perspective. Open Forum Infect Dis. (2022) 9:ofac124. doi: 10.1093/ofid/ofac124 35493113 PMC8992234

[22] Kim YC Kim B Son NH Heo N Nam Y Shin A. Vaccine effect on household transmission of omicron and Delta SARS-CoV-2 variants. J Korean Med Sci. (2023) 38:e9. doi: 10.3346/jkms.2023.38.e9 36593690 PMC9807772

[23] de Gier B Andeweg S Backer JA Hahné SJ van den Hof S de Melker HE. Vaccine effectiveness against SARS-CoV-2 transmission to household contacts during dominance of Delta variant (B.1.617.2), the Netherlands, august to September 2021. Euro Surveill. (2021) 26:2100977. doi: 10.2807/1560-7917.ES.2021.26.44.2100977, PMID: 34738514 PMC8569927

[24] de Gier B Andeweg S Joosten R Ter Schegget R Smorenburg N van de Kassteele J. Vaccine effectiveness against SARS-CoV-2 transmission and infections among household and other close contacts of confirmed cases, the Netherlands, February to May 2021. Euro Surveill. (2021) 26:2100640. doi: 10.2807/1560-7917.ES.2021.26.31.2100640, PMID: 34355689 PMC8343550

[25] Bigelow BF Saxton RE Flores-Miller A Mostafa HH Loss MJ Phillips KH. Community testing and SARS-CoV-2 rates for Latinxs in Baltimore. Am J Prev Med. (2021) 60:e281–6. doi: 10.1016/j.amepre.2021.01.005 33775510

[26] Oh DL Meltzer D Wang K Canchola AJ DeRouen MC McDaniels-Davidson C. Neighborhood factors associated with COVID-19 cases in California. J Racial Ethn Health Disparities. (2023) 10:2653–62. doi: 10.1007/s40615-022-01443-y, PMID: 36376642 PMC9662780

[27] Rozenfeld Y Beam J Maier H Haggerson W Boudreau K Carlson J. A model of disparities: risk factors associated with COVID-19 infection. Int J Equity Health. (2020) 19:126. doi: 10.1186/s12939-020-01242-z 32727486 PMC7387879

[28] Cohen-Cline H Li H-F Gill M Rodriguez F Hernandez-Boussard T Wolberg H. Major disparities in COVID-19 test positivity for patients with non-English preferred language even after accounting for race and social factors in the United States in 2020. BMC Public Health. (2021) 21:2121. doi: 10.1186/s12889-021-12171-z 34794421 PMC8600352

[29] Jimenez ME Rivera-Núñez Z Crabtree BF Hill D Pellerano MB Devance D. Black and Latinx community perspectives on COVID-19 mitigation Behaviors, testing, and vaccines. JAMA Netw Open. (2021) 4:e2117074. doi: 10.1001/jamanetworkopen.2021.17074 34264327 PMC8283554

[30] Al-Kuwari MG Al-Nuaimi AA Abdulmajeed J Semaan S Al-Romaihi HE Kandy MC. COVID-19 infection across workplace settings in Qatar: a comparison of COVID-19 positivity rates of screened workers from march 1st until July 31st, 2020. J Occup Med Toxicol. (2021) 16:21. doi: 10.1186/s12995-021-00311-5, PMID: 34140020 PMC8210512

[31] Goldman N Pebley AR Lee K Andrasfay T Pratt B. Racial and ethnic differentials in COVID-19-related job exposures by occupational standing in the US. PLoS One. (2021) 16:e0256085. doi: 10.1371/journal.pone.0256085 34469440 PMC8409606

[32] Point2Homes. California demographics. (2023). Available online at: https://www.point2homes.com/US/Neighborhood/CA/San-Ysidro-Demographics.html

[33] Guarienti FA Xavier FAC Ferraz MD Bartelle MB Pasa R Angonese A. Identifying COVID-19 variant through symptoms profile: would it be possible? A rapid review. BMC Infect Dis. 24:1306. 10.1186/s12879-024-10094-9 PMC11566187 39543481

[34] Torabi SH Riahi SM Ebrahimzadeh A Salmani F. Changes in symptoms and characteristics of COVID-19 patients across different variants: two years study using neural network analysis. BMC Infect Dis. (2023) 23:838. doi: 10.1186/s12879-023-08813-9 38017395 PMC10683353

[35] DeWitt M Tjaden A Herrington D Schieffelin J Gibbs M Weintraub W. COVID-19 symptoms by variant period in the North Carolina COVID-19 community research partnership, North Carolina, USA. Emerging Infectious Disease journal. (2023) 29:207–11. doi: 10.3201/eid2901.221111 PMC9796200 36573634

[36] dos Santos PR dos Santos UR de Santana Silva ÍTS Fehlberg HF Ferreira FB Albuquerque GR. Influence of SARS-CoV-2 variants on COVID-19 epidemiological and clinical profiles: a comparative analysis of two waves of cases. Virol J. (2024) 21:260. doi: 10.1186/s12985-024-02538-0 39438927 PMC11515746

[37] Wang RC Gottlieb M Montoy JCC Rodriguez RM Yu H Spatz ES. Association between SARS-CoV-2 variants and frequency of acute symptoms: analysis of a multi-institutional prospective cohort study—December 20, 2020—June 20, 2022. Open Forum Infect Dis. (2023) 10:ofad275. doi: 10.1093/ofid/ofad275 37426947 PMC10327880

[38] Tjaden AH Fette LM Edelstein SL Gibbs M Hinkelman AN Runyon M. Self-reported SARS-CoV-2 vaccination is consistent with electronic health record data among the COVID-19 community research partnership. Vaccines. (2022) 10:1016. doi: 10.3390/vaccines10071016 35891180 PMC9316024

[39] Archambault PM Rosychuk RJ Audet M Bola R Vatanpour S Brooks SC. Accuracy of self-reported COVID-19 vaccination status compared with a public health vaccination registry in Québec: observational diagnostic study. JMIR Public Health Surveill. (2023) 9:e44465. doi: 10.2196/44465 37327046 PMC10278735

