# Supplementary material for: Real-world impact of COVID-19 vaccination, household exposure, and circulating SARS-CoV-2 variants on infection risk and symptom presentation in a U.S./Mexico border community
Source: Front Public Health. 2025 Jun 9;13:1497390. doi: 10.3389/fpubh.2025.1497390 (PMC12183289; doi:10.3389/fpubh.2025.1497390)
Supplement: Supplementary file 1 [file Table_1.docx]

| **Supplement Table 1.** Common Data Elements (CDEs) and study-specific data included in the study questionnaire. |
| --- |
| **CONSENT** |
| Date of Consent |
| I agree to be contacted for future research. |
| I agree to have my genetic code sequenced. |
| County |
| **ABOUT YOU** |
| Date of Identity Collection |
| First Name |
| Last Name |
| Street Address |
| Street Address 2 |
| City |
| State or Territory |
| Zip Code |
| Mobile Phone |
| Home Phone |
| Other Phone |
| Personal Email |
| Other Email |
| Preferred Method of Contact |
| Age calculated as difference between test date and birth date. |
| Are you a San Ysidro Health (SYH) patient? |
| Do you have children under 18 years old? |
| Are your children SYH patients? |
| **DEMOGRAPHICS** |
| Date of Sociodemographic Collection |
| What is your race? Check one or more boxes. |
| Are you of Hispanic, Latino, or Spanish origin? |
| Please select your origin |
| You selected "Other". Please write your response. |
| Do you speak a language other than English at home? |
| What language(s)? |
| You selected "Other". Please write your response. |
| What was your sex assigned at birth on your birth certificate? |
| What terms best express how you describe your gender identity? |
| Which of the following best represents how you think of yourself at this time? |
| Are you currently pregnant? |
| What is the highest level of education you have achieved outside or in the United States? Grades roughly equivalent to years of school. |
| **CURRENT SYMPTOMS** |
| Date of Symptom Collection |
| Have you had any of these symptoms during the past week? |
| Fever or chills |
| Cough |
| Shortness of breath or difficulty breathing |
| Lack of energy or general tired feeling |
| Muscle or body aches |
| Headache |
| New loss of taste or smell |
| Runny nose |
| Feeling sick to your stomach or vomiting, diarrhea |
| Abdominal pain |
| Skin rash |
| Other symptom |
| You selected "Other". Please write your response. |
| **EXPOSURE RISK** |
| Have you had contact with anyone who doesn't live with you who has been diagnosed with COVID-19 in the last 2 weeks? |
| Has anyone who lives with you been diagnosed with COVID-19 in the last 2 weeks? |
| How often have you eaten inside a restaurant in the last 2 weeks? |
| During the last two weeks when you leave your house, how often have you worn a mask that covers your mouth and nose? |
| When in outdoor public spaces (when going to and from a store, at a park, or at the beach) when you are LESS than 6 feet from another person |
| When in outdoor public spaces (when going to and from a store, at a park, or at the beach) when you are FARTHER than 6 feet from another person |
| When taking the bus, train, or ride sharing in an Uber/Lyft |
| When in indoor public spaces (stores, hair salons, or business offices) |
| When indoors at the house of a friend or family member who you do not live with |
| **VACCINATION** |
| Date of Vaccine Acceptance Collection |
| Have you ever received a flu vaccination? |
| Have you received a flu vaccine in the last 6 months? |
| Have you received a COVID-19 vaccine? |
| How many doses of the COVID-19 vaccine have you received? |
| You selected "Other". Please write which vaccine you received? |
| When did you receive your first COVID-19 vaccine dose? |
| Have you received a COVID-19 vaccine booster? (A booster is a third dose of vaccine from Pfizer or Moderna, or a second dose of Johnson & Johnson) |
| Which booster vaccine have you received? |
| When did you receive the booster vaccine? |
| Which of these impacted your access to the COVID-19 vaccine? (Please choose all that apply) |
| You selected" Other". Please write your response. |
| What made getting your COVID-19 vaccine difficult? Please choose all that apply. |
| You selected "Other". Please write your response. |
| Was there anything that made you worried when getting the vaccine? (Please choose all that apply) |
| How likely are you to get an approved COVID-19 vaccine in the next 3 months? |
| What are some of the reasons you have NOT gotten the COVID-19 vaccine? (Select all that apply) |
| You selected "Other". Please write your response. |
| **EMPLOYMENT** |
| Have you, or has anyone in your household, experienced a loss of employment income since the start of the COVID-19 pandemic (March 2020)? |
| We would like to know about what you do -- are you working now, looking for work, retired, keeping house, a student, or something else? |
| You selected "Other". Please write your response |
| Are you considered an essential worker? An essential worker is someone who was required to go to work even when stay at home orders were in place. |
| Would any of these describe where you work? |
| What is the primary kind of health insurance or health care plan that you have now? |
| Did you lose health coverage because of the COVID-19 pandemic? |
| The COVID-19 pandemic may cause challenges for some people, whether they get COVID-19 or not. In the past 6 months, have you or your family experienced any of the below challenges? |
| Getting the health care I need (including for mental health) |
| Having a place to stay/live |
| Getting enough food to eat |
| Having clean water to drink |
| Getting the medicine I need |
| Getting to where I need to go |
| **TESTING** |
| Date of Testing Collection |
| If you were to test positive for COVID-19, would you be able to isolate without losing your job? |
| If you would be exposed to someone with COVID-19, would you be able to quarantine without losing your job? |
| Have you ever been tested for COVID-19? |
| Have you ever tested positive for COVID-19? |
| What month did you first test positive for COVID-19? |
| What year did you first test positive for 2019 COVID-19? |
| What month did you have your most recent COVID-19 test? |
| What year did you have your most 2019 recent COVID-19 test? |
| What was the result of your most recent COVID-19 test? |
| How were you tested for your most recent test? |
| I know where I can get COVID-19 testing in my community. |
| It is easy to get tested for COVID-19. |
| How confident are you that a negative test result means that you do not have COVID-19? |
| How confident are you that a positive test result means that you do have COVID-19? |
| How much do the following encourage you to get tested? |
| Reduce worry that I might have COVID-19. |
| Believe that I was exposed to someone who has COVID-19. |
| To know if I am safe not to give COVID-19 to friends and family. |
| To know if I am safe not to give COVID-19 to anyone I am around. |
| To let my employer know that I am safe to work. |
| To get treated early (if I am positive). |
| How much do the following discourage you to get tested? |
| May experience discomfort from being tested. |
| Even if I don't have it when tested, I can still get COVID-19 later. |
| I don't have COVID-19 symptoms, so I don't need to be tested. |
| If I'm positive, officials will need to contact the people I've been in contact with |
| I don't want to know if I have it |
| Not much they can do for me if I have it. |
| Difficult to get needed healthcare if I have it. |
| I plan to get tested as often as needed |
| If I get a negative test result, it means [check all that apply]: |
| If I get a positive result, it means [check all that apply]: |
| Become sick from COVID-19? |
| Been hospitalized from COVID-19? |
| Died from COVID-19? |
| Have you had COVID-19? |
| What do you think your personal level of risk is for getting sick from COVID-19 |
| Have you been tested for COVID-19 in the last 30 days? |
| Why were you tested? (Select the primary reason for your latest test) |
| You selected "Other". Please write your response. |
| Have you encountered any of the following barriers or problems with testing? (Select all that apply) |
| You selected "Other". Please write your response. |
| Does your employer offer paid time off if you test positive? |
| If you need to isolate due to a positive test or illness, what challenges do you face? |
| You selected "Other". Please write your response. |
| For your previous COVID-19 testing, how long did you wait between scheduling the test and when the test was performed? |
| How did you receive your test results? |
| **SOURCES OF INFORMATION** |
| How much do you trust each of these sources to provide correct information about COVID 19? (Select one response for each row) |
| Your doctor or health care provider |
| Your faith leader |
| Your close friends and members of your family |
| People you go to work or class with or other people you know |
| News on the radio, TV, online, or in newspapers |
| Your contacts on social media |
| The U.S. government |
| The U.S. Coronavirus Task Force |
| **HOUSING** |
| Date of Housing, Employment, and Insurance Collection |
| What best describes your family at home: |
| Are you currently living in transitional housing staying in a shelter, or experiencing homelessness? |
| Do you live in any of these? |
| You selected "Somewhere else". Please write your response. |
| **FAMILY INCOME** |
| In 2019, what was your total household income before taxes? |
| Date of Work PPE and Distancing Collection |
| In your workplace, do you have a place to wash your hands? |
| Does your work require you to be in close contact (i.e., within 6 ft) with others? |
| In your workplace, do you have access to necessary personal protective equipment (PPE)? |
| **MEDICAL HISTORY** |
| Date of Medical History Collection |
| Since the start of the COVID-19 pandemic (March 2020), have you needed to postpone any medical care? |
| Have you ever been diagnosed, either currently or in the past with the following? (Select all that apply) |
| Immunocompromised condition |
| Autoimmune disease |
| Hypertension (HTN, high blood pressure) |
| Diabetes |
| Chronic kidney disease (CKD) |
| Cancer diagnosis and/or treatment within the past 12 months |
| Cardiovascular disease (CVD or heart disease) |
| Asthma |
| Chronic obstructive pulmonary disease (COPD) |
| Other chronic lung disease |
| Sickle Cell Anemia |
| Depression |
| Alcohol or substance use disorder |
| Intravenous drug use |
| Other mental health disorder |
| Other chronic condition |
| **HEALTH STATUS** |
| Date of Health Status Collection |
| How tall are you without shoes? (Select one) |
| feet |
| inches |
| meters |
| centimeters |
| Select the units you wish to report your weight in |
| How much do you weigh without clothes or shoes? |
| If you are currently pregnant, how much did you weigh before your pregnancy? |
| Would you say your health in general is excellent, very good, good, fair, or poor? |
| Do you have a disability that interferes with your ability to carry out daily activities? Examples of daily activities include walking, climbing stairs, shopping, balancing a checkbook, bathing or dressing. |
| **ALCOHOL AND TOBACCO/NICOTINE USE** |
| Date of Alcohol/Tobacco Use Collection |
| In your entire life, have you ever had at least 1 drink of any kind of alcohol, not counting small tastes or sips? |
| How often do you have a drink containing alcohol? |
| Do you now smoke cigarettes? |
| If you smoke every day, on average, how many cigarettes per day do you smoke? |
| Do you now use electronic cigarettes every day, some days, rarely, or not at all? |
|  |
|  |
|  |
|  |
|  |
|  |
